# Prenatal diagnosis of a 5q35.3 microduplication involving part of the *ADAMTS2* locus: a likely benign variant without apparent phenotypic abnormality: Case series

**DOI:** 10.1097/MD.0000000000018258

**Published:** 2019-12-10

**Authors:** Fagui Yue, Yang Yu, Qi Xi, Hongguo Zhang, Yuting Jiang, Shibo Li, Ruizhi Liu, Ruixue Wang

**Affiliations:** a Center for Reproductive Medicine, Center for Prenatal Diagnosis, First Hospital; b Jilin Engineering Research Center for Reproductive Medicine and Genetics, Jilin University, Changchun, China; c Department of Pediatrics, University of Oklahoma Health Sciences Center, Oklahoma City, OK.

**Keywords:** 5q35.3 microduplication, part of *ADAMTS2*, SNP array

## Abstract

**Rationale:**

Chromosomal duplications are associated with a series of genetic disorders. However, chromosome 5q duplications, especially pure 5q35.3 microduplications, have rarely been reported in the literature. Clinical phenotypes usually depend on the region of chromosome duplicated, its size, and loci.

**Patient concerns:**

From 2011 to 2017, prenatal amniotic fluid samples were obtained from 6 pregnant women diagnosed with pure 5q35.3 microduplications following different prenatal indications at our center. We followed up the children of these pregnancies and determined their postnatal health conditions.

**Diagnoses:**

Cytogenetic studies delineated that all patients had normal karyotypes, except for patient 6 who had 46,XX,inv(9)(p11q13). Single-nucleotide polymorphism array results showed 177–269 kb duplications of 5q35.3 (chr5:178728830–178997692) in these cases. All shared similar localization of *ADAMTS2*.

**Interventions:**

All pregnant women chose to continue the pregnancies. Follow-up analysis showed that the children presented normal physical and growth developments.

**Outcomes:**

We described six prenatal cases with similar 5q35.3 duplications involving part of the *ADAMTS2* locus with no apparent postnatal phenotypic abnormalities.

**Lessons:**

Our research revealed that partial microduplication of *ADAMTS2* (chr5:178728830–178997692) might be benign and not correlate with disorders. And there might exist phenotypic diversities of 5q35.3 duplications.

## 1 Introduction

Chromosomal duplications are commonly associated with intellectual disability (ID) and related disorders. Three main common manifestations of 5q duplications have been seen in the clinic: distal 5q, interstitial, and terminal duplications,^[1–3]^ whereas Rodewald et al^[4]^ divided 5q duplications into: the proximal duplication of 5q11-q22.1, the distal duplication of 5q31-qter, and the distal duplication of 5q34-qter. Other 5q duplication cases, such as 5q34 and 5q35, have also recently been described.^[5]^

Pure partial 5q duplications are rare, and coverage regions range across the long arm. 5q duplications often occur in association with other chromosomal deletions, which makes it difficult to establish phenotype–karyotype correlations. Two explanations have been put forward to explain the common formations: an unbalanced segregation of a parental balanced translocation between 5q and another chromosome, and a homologous chromosome producing a partial monosomy of 5p and trisomy of 5q resulting from a parental inversion on chromosome 5. The parental insertion or direct duplication can also result in a pure 5q duplication.^[6]^ Based on this information, it is not easy to determine the phenotypes of pure 5q duplications in the clinic.

For submicroscopic chromosomal imbalances and copy number variations (CNVs), the G-banding technique is not suited to detecting structural aberrations; however, chromosomal microarray analysis (CMA) can improve the detection yield because of its superior diagnostic resolution. CMA is performed either as array comparative genomic hybridization or single-nucleotide polymorphism array. Subchromosomal abnormalities in approximately 1% of structurally normal pregnancies and 6% with structural abnormalities can be diagnosed by CMA of prenatal samples with normal karyotypes.^[7,8]^ It can also be used as a postnatal diagnostic tool for children with congenital abnormalities, developmental retardation, and intellectual disability.^[9]^

Here, we delineated 6 cases of prenatal diagnostic 5q35.3 duplication presenting with a normal phenotypic spectrum using the single-nucleotide polymorphism (SNP) array. We also reviewed related clinical data focusing on similar duplicated segments, and discussed the potential pathogenicity of a 5q35.3 microduplication involving part of the ADAMTS2 locus.

## 2 Subjects and methods

### 2.1 Subjects

From 2011 to 2017, prenatal amniotic fluid samples were obtained from 6 pregnant women diagnosed with pure 5q35.3 microduplications following different prenatal indications at the Center for Reproductive Medicine and Center for Prenatal Diagnosis of the First Hospital of Jilin University. We followed up the children of these pregnancies and determined their postnatal health conditions. The study protocol was approved by the Ethics Committee of the First Hospital of Jilin University (No.2011–042), and all patients’ parents had provided informed consent for publication of the cases. All experiments in our study, including cytogenetic analysis and molecular cytogenetics, were performed in accordance with relevant guidelines and standard protocols.

### 2.2 Cytogenetic analysis

Chromosome analysis was performed on G-band metaphases prepared from cultured amino fluid cells according to standard protocols. Twenty metaphases were analyzed for all samples. The International System for Human Cytogenetic Nomenclature (ISCN 2013) was used to describe the karyotype.^[10]^

### 2.3 SNP array

SNP array analysis was performed using the Human CytoSNP-12 BeadChip (Illumina, San Diego, CA). DNA was extracted from 10 mL of uncultured aminotic fluid cells using the QIAamp DNA Mini kit (Qiagen, Hilden, Germany) according to the manufacturer's instructions. Image data were analyzed using Illumina's Genome Studio software. The final results were analyzed using the Database of Chromosomal Imbalance and Phenotype in Humans using Ensemble Resources (DECIPHER), the database of genomic variants, Online Mendelian Inheritance in Man (OMIM), and the National Center for Biotechnology Information. Parents provided 5 mL of peripheral blood which was collected using a standard vacuum extraction blood-collecting system containing EDTA and heparin. Genomic DNA was then isolated from whole blood using the QIAamp DNA Mini kit (Qiagen) following the manufacturer's instructions.

## 3 Results

From 2011 to 2017, a total of 6 cases with pure 5q35.3 duplications were initially detected by SNP array. All cases shared the similar duplication of part of the *ADAMTS2* locus (chr5: 178728830–178772431). The distributions of indications for prenatal diagnosis were as follows: advanced maternal age (5/6), circular of umbilical cord (3/6), Down syndrome risk (2/6), cervical lymphatic hygroma in the fetus (1/6), abnormal childbearing history (1/6), and early embryonic death in previous pregnancies (1/6). Routine cytogenetic analysis showed that all fetuses had normal karyotypes except for P6. Cytogenetic, SNP array, and clinical findings of all cases are summarized in Table 1. The parents of fetuses P2 and P3 chose to undergo SNP array analysis themselves based on the abnormal microarray results of their fetuses. Both fetuses were found to have inherited their 5q35.3 duplications from their paternal side. Neither of the 2 fathers presented with physical or developmental abnormalities. Among the 6 fetuses, the smallest duplication was 177 kb, whereas the largest was 269 kb.

**Table 1:**
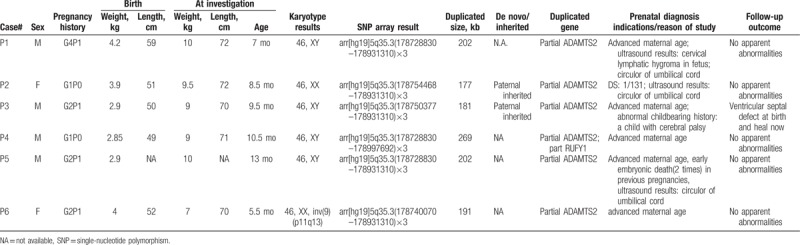
Summary of the cytogenetic, SNP array, and clinical findings of our cases with 5q35.3 duplication.

P1 and P5 shared the same duplicated localizations, although they had different prenatal indications. We then carried out a follow-up of the fetuses in childhood including: pregnancy ultrasound results, body stature, developmental retardation, intellectual disability, craniofacial dysmorphisms, and skeletal anomalies. Our findings are listed in Table 1.

## 4 Discussion

We presented 6 rare prenatal cases with pure 5q35.3 duplications ranging from 177 to 269 kb according to SNP array. Two of these were identified as arising from paternal origins. To the best of our knowledge, the pathogenicity of duplicated CNV has not previously been described before. This is also the first report focusing on pure 5q35.3 duplications with no apparent phenotypic abnormalities. Moreover, most reported distal duplication of chromosome 5q is distributed between 5q35.2 and 5q35.3, and seldom involves single 5q35.3 duplications.^[3]^

Chromosomal rearrangements such as duplications can result in a series of genetic diseases.^[11]^ Indeed, partial duplication of distal chromosome 5q is associated with a wide range of clinical phenotypes including short stature, growth and mental retardation, microcephaly, skeletal anomalies, facial clefts, micrognathia, low-set ears, hypertelorism, almond-shaped eyes, down-slanted palpebral fissures, strabismus, epicanthal folds, a prominent widened nasal bridge, a long philtrum, small mouth, and thin upper lip. Other unusual characteristics include heart defects (ventricular septal defects, atrial septal defects, and a bicuspid aortic valve), hypoplastic phalanges, ambiguous genitalia, hypospadias, cryptorchidism, and inguinal hernias.^[4,12–15]^

Because of the limited number of reported cases, clinical manifestations involving 5q35.3 microduplications only appear to be associated with short stature and microcephaly.^[3]^ Therefore, to delineate the phenotype–karyotype correlations more clearly, we summarized the clinical manifestations of patients involving or overlapping 5q35.3 duplications in Table 2.^[3,14–21]^ The age of the patients ranged from 2 years 8 months to 39 years. Most duplications were located in the region of 5q35.2q35.3 (15/18), with the remainder in 5q35.3 (3/18). The duplicated region ranged from 0.26 to 6.4 Mb. Among the duplications, 6 of 18 patients were *de novo*, 4 of 18 were maternally inherited, and 8 of 18 patients were not available. The high incidence rate of clinical characteristics was as follows: short stature (16/18), growth retardation (15/18), microcephaly (14/18), intellectual disability (14/18), language retardation (12/18), and motor delay (11/18), which is consistent with previous reports of 5q duplications.^[4,12–15]^ Facial dysmorphisms are also important common manifestations for these patients and included: flat philtrum (12/18), long face (11/18), prominent nasal tip (11/18), thin upper lip (11/18), periorbital fullness (9/18), down-slanting palpebral fissures (9/18), strabismus (6/18), low-set ears (5/18), epicanthic folds (5/18), downturned mouth (4/18), a prominent nasal bridge (3/18), and hypertelorism (2/18). The irregular bone age was as follows: delayed (3/18), normal (2/18), and advanced (1/18). Mood swings and autistic symptoms were also reported in these patients (6/18).

**Table 2:**
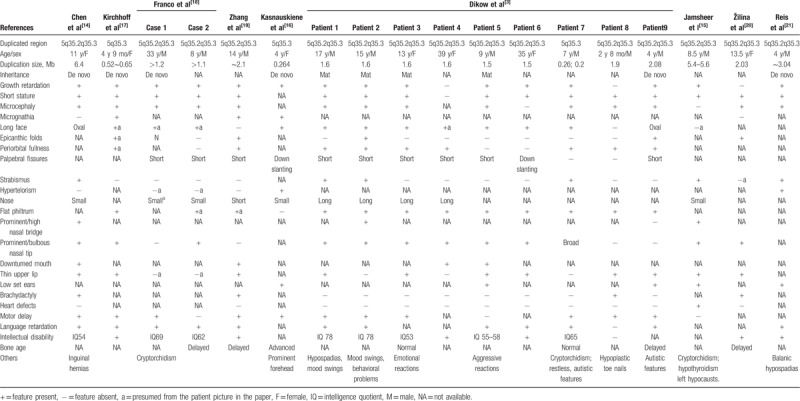
Clinical data of patients involving/overlapping 5q35.3 duplication.

**Figure 1:**
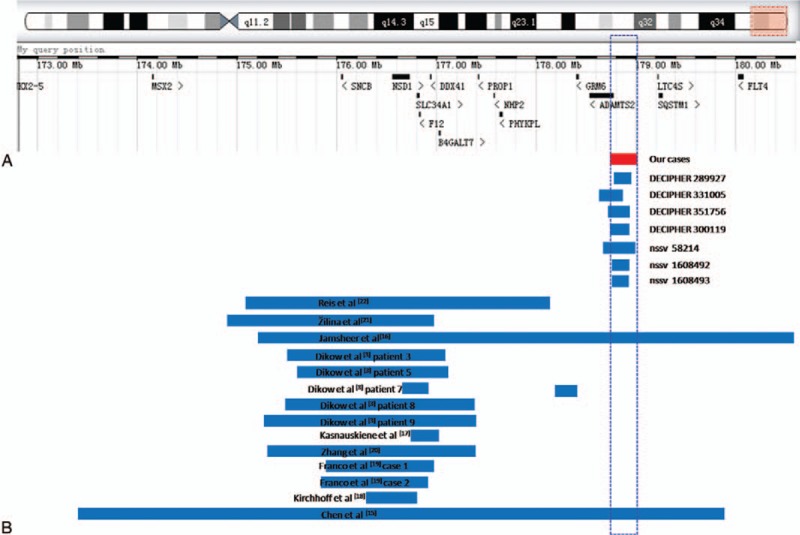
Scale representation of the duplicated region in the distal long arm of chromosome 5 (5q35.2–5q35.3) (https://decipher.sanger.ac.uk/) (A) Location of morbid genes in the region. (B) Duplicated fragments in the present cases (red) and previously described duplications in the region (blue).

Seven cases with similar overlapping 5q35.3 duplications are described in DECIPHER (patients 289927, 331005, 351756, and 300119) and ISCA databases (patients nssv 58214, nssv1608492, and nssv1608492) (Fig. 1). Patients 289927, 331005, and 351756 had a 16p13.2 microduplication, 5q35.3 microdeletion, and 7q36.1 microduplication, respectively, as well as the 5q35.3 duplication. In contrast, patient 300119 had a pure 5q35.3 duplication, presenting with truncal obesity, an abnormal facial shape, autistic behavior, and global developmental delay. Patient nssv 58214 presented with abnormalities of the skeletal system, a cleft upper lip, and Duane anomaly whose pathogenicity is uncertain. However, the pathogenicity of the duplicated region in the other 2 cases (nssv1608492 and nssv1608493) is largely benign, with the manifestation of developmental delay and/or other notable developmental or morphological phenotypes. Therefore, the pathogenicity of these duplicated regions is still uncertain.

Considering the follow-up outcomes of our patients, all children presented with normal growth and mental development, and there were no limb or facial feature anomalies. This differs from previous findings. P3 presented with a ventricular septal defect at birth, which was the only transitory symptom after birth. To further demonstrate the diverse clinical phenotypes, we made detailed comparisons of the cases encompassing the 5q35.2q35.3 duplication (Fig. 1).

According to the DECIPHER database, a total of 13 morbid genes exist in the 5q35.3 region (Table 3), which are associated with a diverse range of clinic phenotypes. The gene dosage effect appears to be involved in some abnormal clinical phenotypes.^[22]^ Our cases all share similar partial duplications of *ADAMTS2* (OMIM 604539; chr5:178728830–178772431) (Fig. 1). *ADAMTS2* contains 22 exons and is a member of the *ADAMTS* gene family. It encodes an enzyme that excises the *N*-propeptide of type I and type II procollagens. *ADAMTS2* haploinsufficiency is associated with the dermatosparaxis type of Ehlers–Danlos syndrome (EDS; OMIM 225410) which is inherited in an autosomal recessive manner.^[23–25]^ EDS is a heterogeneous group of disorders that affect the fragility of soft connective tissues, leading to hypermobile joints and hyperextension of the skin and other organs and tissues. The dermatosparaxis type of this syndrome is characterized by extreme skin fragility, characteristic craniofacial features, easy bruising, and growth retardation.^[26,27]^

**Table 3:**
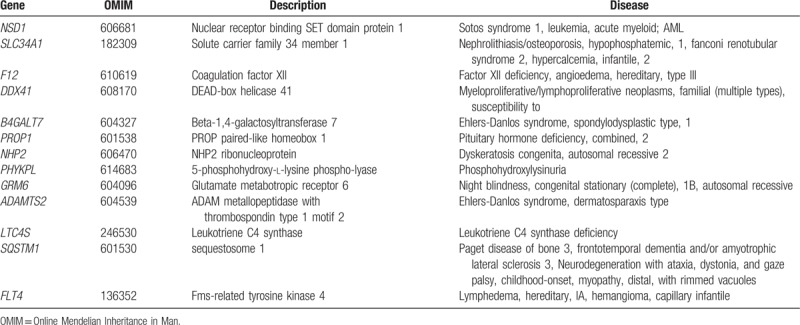
Genes in the region of 5q35.3 and the associated diseases.

There is currently no available evidence for the triplosensitivity in association with *ADAMTS2*. Moreover, P2 and P3 separately inherited the 5q35.3 duplication from their father, presenting without abnormal clinical phenotypes. Therefore, we speculate that the partial duplication of 5q35.3 (chr5: 178728830–178997692), including part of *ADAMTS2*, might be a benign variant. A partial *RUFY1* duplication was also seen in P4; this gene might be involved in endolysosomal transport, which plays an important role in the development of Alzheimer disease.^[28]^ It is therefore likely that this gene has no evident relevance with our subject.

Our research has some limitations. First, the age of individuals at follow-up was typically too young to assess prospective growth and development, especially regarding language and intellectual disability. Table 2 shows that the youngest age reported in previous studies was 2 years 8 months, whereas our youngest subject was 13 months’ old; therefore, regular follow-up for our cases should be carried out in the future. Second, only 2 of the present couples chose to investigate whether their chromosomal duplications are inherited, which is not sufficient evidence to explain pathogenicity of the duplicated region. Additionally, because few reports of similar 5q35.3 duplications have been made previously, further research is needed to confirm our findings.

## 5 Conclusion

We analyzed 6 prenatal cases with similar 5q35.3 microduplications ranging from 177 to 269 kb involving part of the *ADAMTS2* locus by SNP array. The application of molecular cytogenetic techniques provides an effective approach to improve the diagnosis rate of chromosomal microrearrangements and locate functional genes. Our report revealed that the partial 5q35.3 duplication (chr5: 178728830–178997692) might be benign and have no association with human disorders. However, the children involved in this study should undergo an assessment of their growth and intelligence during childhood and adulthood. All cases in our report currently present with normal physical development and clinical manifestations, with no apparent phenotypic abnormalities. This suggests the existence of phenotypic diversities associated with 5q duplications.

## Author contributions

**Conceptualization:** Hongguo Zhang, Shibo Li, Ruixue Wang.

**Data curation:** Qi Xi.

**Formal analysis:** Qi Xi, Yuting Jiang.

**Funding acquisition:** Ruizhi Liu.

**Investigation:** Fagui Yue, Yang Yu, Yuting Jiang, Ruixue Wang.

**Methodology:** Qi Xi, Yuting Jiang, Shibo Li.

**Project administration:** Hongguo Zhang, Ruixue Wang.

**Software:** Yuting Jiang.

**Supervision:** Yang Yu, Hongguo Zhang, Shibo Li, Ruizhi Liu.

**Validation:** Yang Yu, Ruizhi Liu, Ruixue Wang.

**Writing – original draft:** Fagui Yue.

**Writing – review & editing:** Ruixue Wang.
